# Nutrition, frailty, and Alzheimer's disease

**DOI:** 10.3389/fnagi.2014.00221

**Published:** 2014-08-26

**Authors:** Francesco Panza, Vincenzo Solfrizzi, Michele Giannini, Davide Seripa, Alberto Pilotto, Giancarlo Logroscino

**Affiliations:** ^1^Neurodegenerative Disease Unit, Department of Basic Medicine, Neuroscience, and Sense Organs, University of Bari Aldo MoroBari, Italy; ^2^Department of Clinical Research in Neurology, University of Bari Aldo Moro, “Pia Fondazione Cardinale G. Panico”Lecce, Italy; ^3^Geriatric Unit, Laboratory of Gerontology and Geriatrics, Department of Medical Sciences, IRCCS “Casa Sollievo della Sofferenza”San Giovanni Rotondo, Foggia, Italy; ^4^Geriatric Medicine-Memory Unit, Rare Disease Centre, Department of Interdisciplinary Medicine, University of Bari Aldo MoroBari, Italy; ^5^Geriatrics Unit, Azienda ULSS 16 Padova, Hospital S. AntonioPadova, Italy

**Keywords:** frailty and cognitive impairment, nutritional status, Alzheimer's disease, cognition disorders, dementia

Currently, available drugs for the treatment of Alzheimer's disease (AD) have only symptomatic effects, and there is an unmet need of preventing AD onset and delaying or slowing disease progression in absence of disease-modifying therapies. Substantial epidemiological evidence suggested the hypothesis that modifiable metabolic, vascular, and lifestyle-related factors may be linked to the development of late-life cognitive disorders (Solfrizzi et al., 2011). Among these proposed factors, one appealing link is the association between dietary habits and the occurrence of AD (Tangney, 2014).

Among age-related conditions closely associated to dementia and cognitive disorders in late life, frailty is a multidimensional geriatric condition that reflects a multisystem physiological change and a nonspecific state of vulnerability (Fried et al., 2001), with an increased risk for different adverse health outcomes in older age, including disability, falls, hospitalizations, and all-cause mortality (Fried et al., 2001). Although the operational definition of frailty is still controversial, in general, two approaches predominate. The “phenotypic” or physical definition of frailty or the “biological syndrome model” proposed five components: exhaustion, unintentional weight loss, weakness, slow walking speed, and low levels of physical activity. The frail state is defined by the presence of three or more of these components, the pre-frail state is defined by only one or two of these characteristics, while older individuals are “robust” when they have none of these frailty components (Fried et al., 2001). Other definitions, criticizing this concept, promote a multidimensional approach with a definition of frailty based on a cumulative model, employing frailty indexes for the evaluation of this condition, calculated by considering the accumulation of potential deficits, i.e., the presence of diseases, abnormal laboratory values, symptoms, signs, or disabilities (Rockwood et al., 2004). For all frailty models, cognitive and affective disorders, physical activity, and nutritional status have been suggested as markers of frailty (Kelaiditi et al., 2014). In particular, cognition has been considered a major component of frailty, also associated with adverse health outcomes (Rockwood et al., 2004). Therefore, possible preventive interventions on cognitive-related outcomes of frailty, including AD and dementia, may be operated though the prevention of this geriatric syndrome and its associated components (Panza et al., 2011; Robertson et al., 2013). Of note, in frailty prevention, the impaired nutrition, and weight loss of frail older subjects may be addressed by focused nutritional interventions.

## Dietary patterns, Alzheimer's disease, and late-life cognitive disorders

A growing body of evidence suggested that particular diets have been linked to a lower incidence of AD and late-life cognitive disorders (Tangney, 2014). In fact, dietary-related factors, affecting cardiovascular risk, may also influence dementia risk (Solfrizzi et al., 2011). In particular, the Dietary Approaches to Stop Hypertension (DASH) diet, originally developed as a plant-focused dietary plan against hypertension, considers high consumption of vegetables, fruits, lean meats, fish, nuts, legumes, poultry, whole grains, and low-fat dairy products, and lower intake of sodium, red meat, saturated fats, and sweets. In a randomized clinical trial (RCT), higher levels of accordance with the DASH diet conferred greater cognitive improvements in comparison to control subjects (Smith et al., 2010). Higher adherence to the DASH diet demonstrated also to lower the risk of incident dementia over 6 years of follow-up in a population-based study (Norton et al., 2012). In an 11-years follow-up, higher adherence to both the DASH diet and a Mediterranean-style dietary pattern in older age was associated with a significant reduction in rates of global cognitive decline in the same population-based setting (Wengreen et al., 2013).

The Mediterranean diet (MeDi) is another plant- focused dietary pattern with higher intake of vegetables, fruits, breads, other forms of cereals, nuts, potatoes, legumes, and seeds, with a low-to-moderate consumption of fish, poultry, red meat, and wine, while olive oil is the main source of monounsaturated fatty acids (MUFA). In several recent population-based studies, higher levels of accordance with a Mediterranean-type diet has been linked to slower cognitive decline, reduced risk of AD, transition from mild cognitive impairment (MCI) to AD, and decreased mortality in AD patients (Solfrizzi et al., 2011; Tangney, 2014). Furthermore, the results of these population-based studies were confirmed by very recent systematic reviews and meta-analyses showing that adherence to the MeDi was related with a reduced risk of cognitive impairment and decline, MCI, AD, and progression from MCI to AD (Psaltopoulou et al., 2013; Singh et al., 2014). In a recent RCT with a 6.5-years follow-up, nutritional intervention with MeDi enhanced with both extra-virgin olive oil or mixed nuts appeared to improve global cognition after adjustment for possible confounders (Martínez-Lapiscina et al., 2013). This body of evidence confirmed that a MeDI-based nutrient/food model characterized by higher intake of MUFA, n-3 polyunsaturated fatty acids, fish, high levels of antioxidants from fruit and vegetables, and low-to-moderate alcohol consumption may be protective against AD and dementia (Solfrizzi et al., 2011).

## Proposed definition of cognitive frailty as a new clinical entity in older age

Epidemiological evidence strongly suggested that physical frailty may be linked to cognitive impairment (Solfrizzi et al., 2012) and decline in late life (Samper-Ternent et al., 2008), incident AD (Buchman et al., 2007), MCI (Boyle et al., 2010), vascular dementia (VaD) (Avila-Funes et al., 2012; Solfrizzi et al., 2013), non-AD dementias (Gray et al., 2013), and AD pathology in older subjects with and without a diagnosis of dementia (Buchman et al., 2008) (Table 1). For frailty operationalized with the deficit accumulation and multidimensional approach, three Canadian studies, using the population-based sample of the Canadian Study of Health and Aging, found that different measures of frailty at baseline were related with cognitive decline over a 5-years period (Mitnitski et al., 2011a,b) and with dementia and AD over 5- and 10-year intervals (Song et al., 2011) (Table 1).

**Table 1:** **Principal population-based studies on the association of deficit accumulation/multidimensional operational definition of frailty, frailty instruments, and physical/phenotypic/biological operational definition of frailty with late-life cognitive impairment and decline, mild cognitive impairment (MCI), dementia, Alzheimer's disease (AD), and other different cognitive outcomes**.

**References**	**Study design and sample**	**Frailty and cognitive assessment**	**Principal results**
Buchman et al., 2007	Population-based, longitudinal study (3 years); 823 older persons (mean age: 80.4 years) without dementia who participated in the Rush Memory and Aging Project	Physical frailty phenotype operalizionated slightly modifying the CHS criteria and based on four frailty components. Diagnoses of AD and DLB were made according to the NINCDS-ADRDA and the Report of the Consortium on DLB International Workshop. The MMSE was used to describe the cohort, while scores on other 19 neuropsychological tests were used to create a composite measure of global cognitive function. The CIM was used for diagnostic classification purposes only	Both baseline level of frailty and annual rate of change in frailty were associated with an increased risk of incident AD. Furthermore, the level of frailty and rate of change in frailty were also associated with the rate of cognitive decline
Buchman et al., 2008	Population-based, longitudinal study; brain autopsies from 165 deceased participants from the Rush Memory and Aging Project	Physical frailty phenotype operalizionated slightly modifying the CHS criteria and based on four frailty components. Diagnoses of AD and DLB were made according to the NINCDS-ADRDA and the Report of the Consortium on DLB International Workshop criteria. Neuropathological measures of AD pathology, Lewy bodies, and cerebral infarcts were also obtained	Physical frailty proximate to death was related to level of AD pathology on post-mortem examination but was not related to the presence of cerebral infarcts or Lewy body disease. This association was similar in persons with and without dementia
Samper-Ternent et al., 2008	Population-based, longitudinal study (10 years); 1370 non-institutionalized Mexican American men and women aged 65 years and older from the H-EPESE with a MMSE ≥ 21 at baseline	Physical frailty phenotype operalizionated slightly modifying the CHS criteria based on four frailty components and excluding physical activity and MMSE	A statistically significant association between frailty and subsequent decline in cognitive function over a 10-years period was found in older Mexican Americans
Avila-Funes et al., 2009	Population-based, cross-sectional and longitudinal study (4 years); 6030 older individuals aged 65–85 years from the Three-City Study	Physical frailty phenotype operalizionated slightly modifying the CHS criteria, MMSE, and IST. Diagnosis of dementia according to the DSM-IV criteria	Frail individuals with cognitive impairment have a higher risk of IADL and ADL disability and of incident hospitalization and dementia than subjects with none of these conditions, even after adjusting for potentially confounding variables
Boyle et al., 2010	Population-based, longitudinal study (12 years); 761 older persons (mean age: 79 years) without cognitive impairment at baseline who participated in the Rush Memory and Aging Project	Physical frailty phenotype operalizionated slightly modifying the CHS criteria and based on four frailty components. Diagnoses of AD and MCI were made according to the NINCDS-ADRDA criteria and CSHA clinical criteria. The MMSE was used to describe the cohort, while scores on other 19 neuropsychological tests were used to create a composite measure of global cognitive function. The CIM was used for diagnostic classification purposes only	Higher level of physical frailty predicted the development of MCI and is associated with an accelerated rate of cognitive decline in older persons
Mitnitski et al., 2011a	Population-based, longitudinal study (5 years); 9266 individuals of the CSHA sample subjects aged 75.8 ± 7.1 years	CSHA Frailty Index and 5-years change in errors on 3MS grouped into categories of 3	Frailty at baseline associated with cognitive change in men and women
Mitnitski et al., 2011b	Population-based, longitudinal study (5 years); 2305 individuals from the CSHA sample of subjects aged 83.1 ± 6.9 years	CSHA Frailty Index, CSHA Clinical Frailty Scale, CHS frailty phenotype and 5-years change in errors on 3MS grouped into categories of 3	All measures of frailty at baseline associated with cognitive decline
Song et al., 2011	Population-based, longitudinal study (5 and 10 years); 5909 individuals from the CSHA aged 65 years and older	Frailty Index of “non-traditional” risk factors for dementia and diagnosis of dementia according to the DSM-III-R and diagnosis of AD according to NINCDS-ADRDA criteria	Frailty at baseline was associated with the incidence of AD and dementia of all types over 5- and 10-year intervals
Solfrizzi et al., 2012	Population-based, longitudinal study (3 and 7 years); 2581 individuals from the ILSA sample of 5632 subjects aged 65–84 years	Physical frailty phenotype operalizionated slightly modifying the CHS criteria and diagnosis of dementia according to the DSM-III-R, NINCDS-ADRDA, and ICD-10 criteria	Lower cognition was associated with physical frailty. Frail demented patients were at higher risk of all-cause mortality over 3- and 7-years follow-up periods, but not of disability
Cano et al., 2012	Population-based, longitudinal study (10 years); 1815 Mexican American men and women aged 67 years and older from the H-EPESE	Physical frailty phenotype operalizionated slightly modifying the CHS criteria and MMSE > 21	As MMSE score declined over time, the percent of frail individuals increased in a linear fashion. Frailty and cognitive impairment are independent risk factors for mortality after controlling for all covariates. When both cognitive impairment and frailty were added to the model, hazard ratio for individuals with cognitive impairment was no longer statistically significant
Avila-Funes et al., 2012	Population-based, longitudinal study (7 years); 5480 older individuals aged 65–85 years from the Three-City Study	Physical frailty phenotype operalizionated slightly modifying the CHS criteria, MMSE, and IST. Diagnosis of dementia according to the DSM-IV criteria and VaD according to the NINDS-AIREN criteria	Frailty was marginally associated with greater risk of all types of dementia and was not associated with incident AD, but frailty status was independently associated with incident VaD
Solfrizzi et al., 2013	Population-based, longitudinal study (3.5 years); 2581 individuals from the ILSA sample of 5632 subjects aged 65–84 years	Physical frailty phenotype operalizionated slightly modifying the CHS criteria and diagnosis of dementia according to the DSM-III-R, NINCDS-ADRDA, and ICD-10 criteria	Over a 3.5-years follow-up, frailty syndrome was associated with a significantly increased risk of overall dementia and, in particular, VaD, while the risk of AD or other types of dementia did not significantly change in frail individuals in comparison with subjects without frailty syndrome
Gray et al., 2013	Population-based, longitudinal study (6.5 years); 2619 individuals from the Adult Changes in Thought (ACT) study sample of subjects aged 65 years and older	Physical frailty phenotype operalizionated with the CHS criteria and diagnosis of dementia according to the DSM-IV, diagnosis of possible and probable AD according to NINCDS-ADRDA criteria. Non-AD dementia consisted of all dementias not classified as possible or probable AD	Frailty was associated with higher risk of developing non-AD dementia but not AD. Although frailty was not associated with all-cause dementia in the entire sample, an association did exist in participants with higher cognitive scores

*CHS, Cardiovascular Health Study; DLB, dementia with Lewy bodies; NINCDS-ADRDA, National Institute of Neurological and Communicative Disorders and Stroke–Alzheimer's Disease and Related Disorders Association; MMSE, Mini Mental State Examination; CIM, Complex Ideational Material; EPESE, Established Population for the Epidemiological Study of the Elderly; IST, Isaac Set Test; DSM-IV, Diagnostic and Statistical Manual of Mental Disorders-IV; IADL, instrumental activities of daily living; ADL, activities of daily living; CSHA, Canadian Study of Health and Aging; 3MS, modified Mini Mental State Examination; DSM-III-R, Diagnostic and Statistical Manual of Mental Disorders-III revised; ICD-10, International Statistical Classification of Diseases and Related Health Problems, 10th revision; ILSA, Italian Longitudinal Study on Aging; VaD, vascular dementia; NINDS-AIREN, National Institute of Neurological Disorders and Stroke-Association Internationale pour la Recherche et l'Enseignement en Neurosciences*.

Very recently, this extensive body of epidemiological evidence linking frailty models to cognition in older age has suggested to an international consensus group of the International Academy of Nutrition and Aging and the International Association of Gerontology and Geriatrics to propose the concept of “cognitive frailty” for describing the simultaneous presence of both physical frailty and cognitive impairment operationalized with a Clinical Dementia Rating scale of 0.5 (i.e., questionable dementia, a stage of this scale similar to MCI in the dementia continuum) in older individuals without dementia concurrent AD or other dementias (Kelaiditi et al., 2013). Of note, cognitive frailty may also represent a precursor of neurodegenerative processes and AD.

In 2006, the term “cognitive frailty” was firstly used to indicate a cognitive vulnerability in MCI and other predementia syndromes exposed to vascular risk and with an increased transition to dementia, particularly VaD (Panza et al., 2006). Therefore, this new clinical entity should be validated as a possible precursor of cognitive-related outcomes, i.e., dementia and its different subtypes. At present, no epidemiological evidence of a progression of cognitive frailty toward dementia is available. However, several studies investigated the possible association of this new complex clinical phenotype with the increased risk of the principal frailty-related adverse health outcomes, with some longitudinal population-based studies reporting an increased risk of disability and all-cause mortality associated to some cognitive frailty models (Avila-Funes et al., 2009; Cano et al., 2012; Solfrizzi et al., 2012; Sampson et al., 2014) (Table 1).

## Nutrition and frailty

Recently, an increasing interest was focused on the impact of several nutritional factors on frailty and its components (Bartali et al., 2006; Ble et al., 2006). In fact, the progression of frailty syndrome has been associated with a more sedentary lifestyle, a reduction in metabolic cell mass, and lower energy expenditure and dietary intake (Inzitari et al., 2011). Lower dietary intake is also linked to the risk of a suboptimal nutritional state or combined micronutrient deficiencies. In fact, undernutrition is among the possible causes of frailty, and older individuals with a treatable condition as protein energy undernutrition may also have poorer cognition (Tamura et al., 2013).

Epidemiological evidence suggested that reduced intake of specific micro- and macronutrients may be associated with reduced caloric intake and weight loss. Different hypotheses have been suggested to explain the mechanisms underlying these associations, including also the reduction of the effects of the antioxidant intake associated with some nutrients. Among macronutrients, higher prevalence of frailty was related to lower protein intake (Bartali et al., 2006). Among micronutrient biomarkers, consumption of higher dietary intake of vitamin C has been associated with frailty-related outcomes (Cesari et al., 2004), while prevalence of frailty was linked to lower intake of vitamin D, E, and C (Bartali et al., 2006). Low serum concentrations of vitamin D, E, and carotenoids were an independent risk factor for frailty among disabled older women, and the risk of frailty appeared to increase with the number of micronutrient deficiencies (Semba et al., 2006). Finally, the association between vitamin D and physical performance showed mixed findings in observational studies and clinical trials (Annweiler et al., 2009).

These findings on the association among micro- and macronutrients and frailty suggested to examine also dietary patterns rather than single nutrients. Over a 6-years period of follow-up, in a population-based setting, higher levels of accordance with a Mediterranean-style diet at baseline was associated with lower risk of incident frailty, and, among frailty components, with lower risk of low walking speed and low physical activity (Talegawkar et al., 2012). Moreover, in a cross-sectional population-based study, higher adherence to MeDi was inversely linked to prevalence of frailty (Bollwein et al., 2013). Adherence to a Mediterranean-style diet was also inversely associated with risk of disability in women, while no association was evidenced in men (Féart et al., 2011). Among frailty components, in community dwelling older persons, higher levels of accordance with a Mediterranean-style diet was associated with a slower mobility decline in an 8 years of follow-up (Milaneschi et al., 2011). Finally, among community dwelling older individuals with higher MeDi adherence at baseline, walking speed over 8 years was faster (Shahar et al., 2012). Taken together these findings may suggest a long-term effect of MeDi also on mobility performance in older age.

Among modifiable factors potentially affecting frailty status in older age, nutrition and physical exercise may be of major relevance (Kelaiditi et al., 2014). In frail older people, some systematic reviews showed that physical exercise interventions may have the potential to improve mobility and functional outcomes (de Vries et al., 2012). Furthermore, impaired nutrition and weight loss typically associated to frail state may be also satisfactorily addressed by specific nutritional interventions. In fact, complete caloric nutritional supplements may produce not only weight gain but also improve cognition as showed in a recent meta-analysis on over1800 older individuals (Allen et al., 2013).

## Conclusion

A healthy diet may have a profound impact on many possible risk factors for AD and cognitive decline, so influencing the onset and progression of these disorders. In particular, healthy dietary models as MeDi or DASH diet may have the potential of modifying some cognitive outcomes, and some recent prospective studies focusing on AD and dementia appeared to be really promising. In fact, higher levels of accordance with the MeDi has been linked to slower cognitive decline, reduced risk of AD, transition from MCI to AD, and decreased mortality in AD patients. Therefore, higher adherence to the MeDi may affect the risk of both MCI and AD, probably influencing also disease progression. However, cognitively intact individuals have increased chance to maintain healthy lifestyle and diet, while more or less mildly cognitively impaired individuals apparently do not. One important task is to test whether lifestyle and dietary interventions may improve cognition to the extent that the individuals in question regain the ability to permanently maintain healthy lifestyle and diet associated with cognitive improvement without intervention.

In the prevention of AD and late-life cognitive disorders, other age-related conditions linked to cognition may be important and modifiable targets. Epidemiological evidence suggested that frailty appeared to be strongly associated with cognitive decline and dementia, while cognitive impairment may also increase the risk of developing frailty suggesting that these two conditions may interact in older age. Therefore, frailty may represent a novel modifiable target in early dementia. The multifactorial etiology of frailty include inflammatory processes, vascular, and hormonal factors, and, among possible determinants, nutritional influences may be of major relevance. Furthermore, AD is also characterized by a multifactorial pathogenesis, requiring interventions acting simultaneously at different levels of the disease, with a multidomain approach and multiple objectives. This approach is also supported by some ongoing RCTs and European initiatives targeting several dementia risk factors in older adults, mainly by promoting lifestyle changes and adherence to medical treatments for vascular diseases and risk factors (Mangialasche et al., 2012). In frailty, impaired nutrition and weight loss may be addressed by specific nutritional interventions. Findings from very recent RCTs showed that physical exercise training in combination with protein supplementation (van de Rest et al., 2014) or alone (Langlois et al., 2013) improved also cognitive outcomes in frail and pre-frail states. Notwithstanding these promising findings, other RCTs are needed to investigate the role of nutrition on late-life cognitive disorders and frailty, so suggesting new viable routes for the prevention and management of cognitive decline and AD in cognitive frail state.

### Conflict of interest statement

The authors declare that the research was conducted in the absence of any commercial or financial relationships that could be construed as a potential conflict of interest.
